# *OsGATA7* modulates brassinosteroids‐mediated growth regulation and influences architecture and grain shape

**DOI:** 10.1111/pbi.12887

**Published:** 2018-02-13

**Authors:** Yan‐Jie Zhang, Yu Zhang, Liang‐Li Zhang, Hui‐Ya Huang, Bao‐Jun Yang, Sheng Luan, Hong‐Wei Xue, Wen‐Hui Lin

**Affiliations:** ^1^ School of Life Sciences & Biotechnology The Joint International Research Laboratory of Metabolic & Developmental Sciences Shanghai Jiao Tong University Shanghai China; ^2^ State Key Laboratory of Plant Molecular Physiology Institute of Botany Chinese Academy of Sciences Beijing China; ^3^ National Key Laboratory of Plant Molecular Genetics CAS Center for Excellence in Molecular Plant Sciences Institute of Plant Physiology and Ecology Shanghai Institutes for Biological Sciences Chinese Academy of Sciences Shanghai China; ^4^ Department of Plant and Microbial Biology University of California Berkeley CA USA

**Keywords:** GATA factor, brassinosteroids, rice architecture, grain shape, grain yield

Rice (*Oryza sativa*) is the most important crop and feeds more than half of the world population. The architecture of rice, including height, leaf inclination and tiller number, is important for rice planting and yield. Plant hormone, brassinosteroids (BR), plays crucial roles in modulating plant architecture and seed yield. But BR cannot be applied in agriculture production directly because BR regulated multiple processes and could not be transcriptional regulated in different tissues. The rice BR‐deficient and BR‐insensitive mutants, such as *d11*,* d2* and *d61*, are dwarf with erect leaves (the ideal architecture for dense planting, Sumiyo *et al*., 2005; Yamamuro *et al*., 2000). But BR deficiency/insensitivity also leads to decreased reproductivity and grain yield. Enhanced BR signalling contributes to better nutrition, higher efficiency of carbohydrate transportation from source to sink and increased grain yield. But the increased plant height and leaf inclination induces lodging and reduces planting density. One of solutions is identifying new regulators which can mediate parts of BR‐regulated rice growth. Here, we identified a new GATA factor, *OsGATA7*, modulates BR‐mediated growth regulation in architecture and grain shape.

GATA factors are a large family of transcriptional regulators found in fungi, animals and plants. All GATA factors feature zinc finger motif (CX2CX17–20CX2C) and the contiguous basic region as DNA binding domain (Reyes *et al*., 2004). There are 28 and 29 putative GATA members in rice and Arabidopsis, respectively. In Arabidopsis, several GATA factors have been studied. *HAN* gene regulates *WUS* expression and boundary formation between meristem and lateral organs. *GNC* and *GNL* affect chloroplast content. Overexpression of *ZIM* leads to elongated hypocotyl and petiole. *NIT2* is involved in nitrate‐dependent control of transcription. And *AtGATA2* functions in light‐ and BR‐regulated photomorphogenesis. In rice, two GATA factors have been functionally characterized. The *Cga1* regulates chloroplast development and plant architecture (Hudson *et al*., 2013) and *NL1* modulates organogenesis during reproductive development (Wang *et al*., 2009).

We identified a rice GATA regulator, *OsGATA7* (Os10g0557600), which was a member of rice GATA subfamily I. There are two exons and one intron in *OsGATA7* genomic DNA, and OsGATA7 protein has one zinc finger/GATA domain located at the C‐terminus. qRT‐PCR analysis revealed the constitutive expression of *OsGATA7* in various tissues, including young/old leaf blade, young/old leaf sheath, leaf lamina joint, young/old root, stem and panicle, with a relatively higher level in young leaf sheath and panicle (Fig. 1a). Studies using transgenic plant harbouring promoter‐GUS vector (pOsGATA7‐GUS) demonstrated the expression pattern of *OsGATA7* in roots, leaves, lamina joint, internodes, lemma and stamens (Fig. 1a).

**Figure 1:**
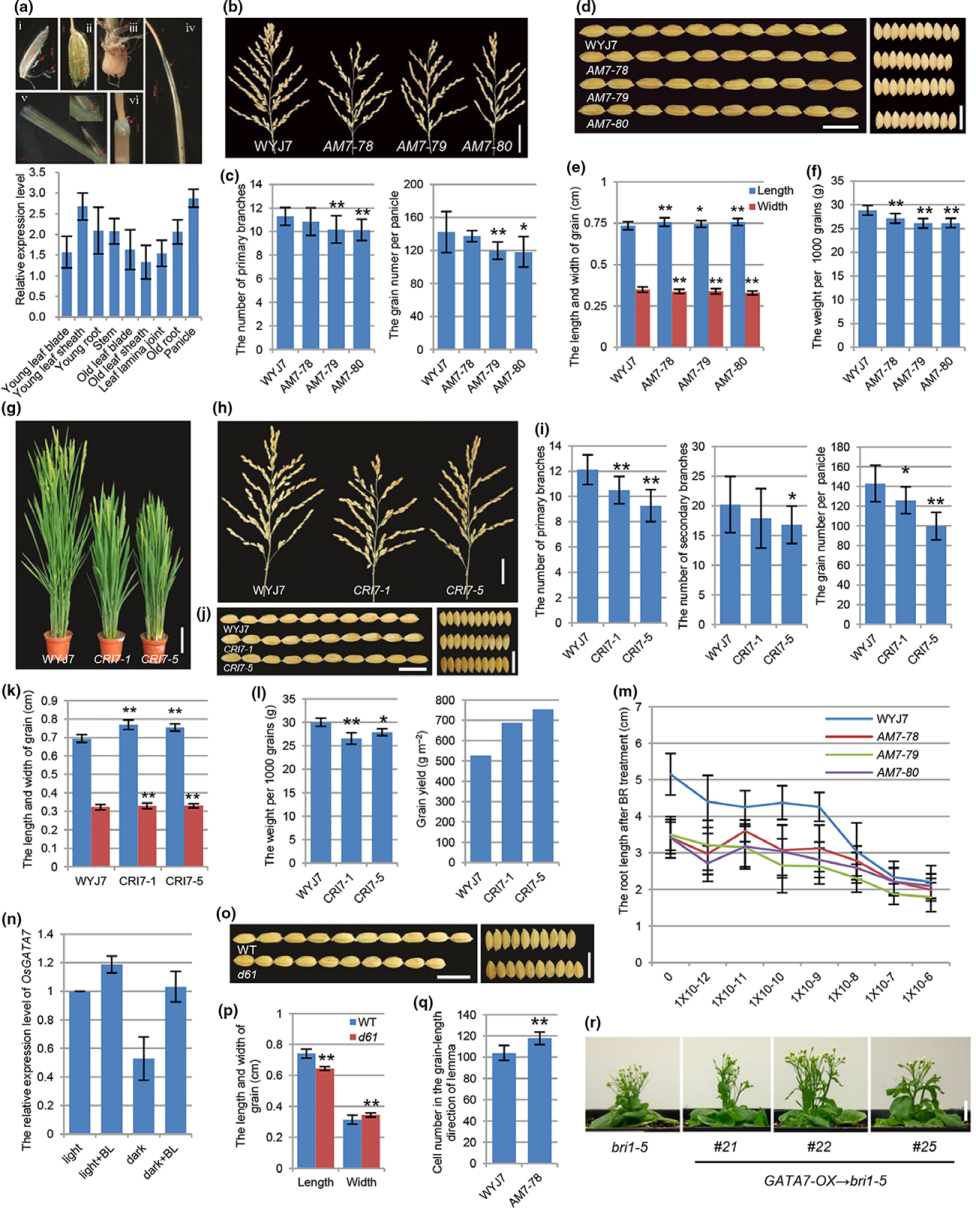
*OsGATA7* modulates BR‐mediated growth regulation in rice. (a) The expression pattern of *OsGATA7*.GUS staining assay of pOsGATA7‐GUS lines shows GUS activity in stamen and pistil (i), lemma (ii), young root (iii), young leaf blade (iv), lamina joint (v), internode (vi). Bars = 2 cm. qRT‐PCR analysis shows the relative expression level of *OsGATA7*. (b) The panicles of WT and AM lines. Bar = 4 cm. (c) Statistical analysis of primary branch number and grain number of WT and AM lines. Values are means ± SE (n = 6–11). **P < 0.01, *P < 0.05. The Student's *t*‐test was used to analyse the significant differences between wild type and AM lines (the same below). (d) The grains of WT and AM lines. Bars = 1 cm. (e) Statistical analysis of grain length and width of WT and AM lines. Values are means ± SE (n = 35–54). **P < 0.01, *P < 0.05. (f) Statistical analysis of weight per 1000 grains. Values are means ± SE (n = 3), **P < 0.01. (g) The adult plants of WT and CRI lines. Bar = 10 cm. (h) The panicles of WT and CRI lines. Bar = 3 cm. (i) Statistical analysis of primary branch number, secondary branch number and grain number per plant. Values are means ± SE (n = 9–29, 9–19 and 8–12). **P < 0.01, *P < 0.05. (j) The grains of WT and CRI lines. Bars = 1 cm. (k) Statistical analysis of grain length and width in WT and CRI lines. Values are means ± SE (n = 27–32). **P < 0.01. (l) Statistical analysis of weight per 1000 grains, grain yield per plant (values are means ± SE, n = 3–6, 9–19, **P < 0.01. *P < 0.05) and the results of grain yield per unit area. (m) Statistical analysis of root length of WT and AM lines after 12‐day eBL treatment. Values are means ± SE (n = 17–20). (n) The relative expression level of *OsGATA7* in 6‐day‐old seedlings after 1 × 10^−6^ M eBL treatment under light and in darkness. (o) The grains of WT and *d61*. Bars = 1 cm. (p) Statistical analysis of grain length and width in WT and *d61*. Values are means ± SE (n = 10). *P < 0.05. (q) Statistical analysis of the outer epidermal cell number in the grain (length) of lemma by scanning electron microscope. Values are means ± SE (n = 10). **P < 0.01. (r) Overexpression of *OsGATA7* in Arabidopsis BR‐insensitive mutant *bri1‐5*. Bar = 1 cm.

To investigate the physiological functions of *OsGATA7*, we constructed transgenic lines using artificial microRNA (AM lines) to knock down the expression of *OsGATA7* in japonica rice WYJ7. qRT‐PCR analysis showed that *OsGATA7* expression was repressed in AM lines. AM lines have decreased height and leaf inclination, as well as reduced primary branch, grain number and grain weights (Fig. 1b, c, and f). Also we constructed genome‐edited lines using CRISPR/Cas9 (CRI lines) technology in WYJ7. Sequencing results illustrated two CRI lines (CRI7‐1 and CRI7‐5) had mutations and generated stop site in the exons. CRI lines have obviously decreased height and leaf inclination (Fig. 1g), illustrating more stunted and compact architecture than wild type (more significantly than AM lines). CRI lines have reduced primary branch, secondary branch (no significant difference in AM lines), grain number and grain weight (Fig. 1h, i and l), similar to AM lines. It seemed that the architecture and grain number/weight of AM and CRI lines are similar to weak alleles of BR‐deficient/insensitive mutants. In addition, the root length of AM seedling is much shorter than WT (Fig. 1m). When treated with eBL (epi‐brassinolide), root growth is less severely inhibited in the AM seedling than WT (Fig. 1m), suggesting AM lines are less sensitive to BR. qRT‐PCR also illustrated BR‐regulated expression of *OsGATA7* (Fig. 1n). Above all, the architecture, the branch/grain number, the grain weight, the BR sensitivity and BR‐regulated gene expression together supposed that *OsGATA7* was involved in BR‐mediated plant growth regulation.

Although *OsGATA7* AM and CRI lines have some phenotypes similar to BR‐deficient/insensitive mutants and show interfered BR signal and response, these lines have better growth condition than reported BR‐related mutants, indicating *OsGATA7* has not as severe influences as BR signalling in rice growth, especially in reproductive development. Even the grain number and weight are slightly lower in individual plant of AM and CRI1 lines, and the selfed‐seed fertilities of CRI1 lines were slightly lower than WT (AM lines were similar to WT), the yield analysis still illustrated that the grain weight of CRI lines was higher (30.4% and 43.3% in CRI7‐1 and CRI‐5 line) than wild type in the same area (Fig. 1l) because of dense planting as these lines have compact architecture. Besides, the grains (as well as brown rice) produced from AM lines (Fig. 1d and e) and CRI lines (Fig. 1j and k) are longer and narrower than WT. Detailed analysis of the outer epidermal cell showed that AM lines had increased cell number (Fig. 1q) but not cell elongation, whereas BR was considered to mainly induce cell elongation. Grain shape is a preference which links to rice quality. Our statistical analysis (Fig. 1o and p) further demonstrated that BR‐insensitive mutant *d61* had shorter and round grain, suggesting that *OsGATA7* played different roles in grain shape regulation with BR signalling. Taken together, *OsGATA7* functions in controlling rice plant architecture and panicle/grain development and the knock‐down/genome‐edited lines have ideal traits of both architecture and grain shape. The CRI lines have enhanced grain yield than WT.

As well as AM and CRI lines, we also constructed the overexpression lines of *OsGATA7* (pUbi‐OsGATA7). There was no significant phenotype in adult plant of overexpression lines, which might be because that the increased *OsGATA7* transcription level in wild type would not be enough to induce growth phenotypes or *OsGATA7* worked with other partners and could not induce growth phenotypes by overexpressing it alone. Many literatures reported Arabidopsis BR‐deficient or BR‐insensitive mutants have phenotypes of dwarf, round leaves, shorter petioles and reduced reproductively. Overexpression of *OsGATA7* could partially rescue phenotypes of Arabidopsis BR‐insensitive mutant *bri1‐5*, such as plant height (Fig. 1r), round leaves and short petioles, but not rosette leaf sides (important for Arabidopsis plant density), which further suggested that *OsGATA7* enhanced BR signalling and partially modulated BR‐mediated plant growth in some processes.

Above all, *OsGATA7* is a multifunction gene regulating rice growth. The plant height, leaf inclination, panicle development and grain number/shape/weight of *OsGATA7* knock‐down/genome‐edited lines suggest *OsGATA7* is involved in BR‐mediated growth regulation. Interestingly, BR positively regulates *OsGATA7* expression, and *OsGATA7* also affects BR signalling and sensitivity, indicating BR and *OsGATA7* synergistically regulate some processes of rice growth. But *OsGATA7* has diverse functions in others processes, such as grain shape regulation, indicating that *OsGATA7* has only partial overlap with BR signalling regulation of rice growth. Partially rescued phenotypes of Arabidopsis *bri1‐5* mutant by *OsGATA7* overexpression further demonstrated that *OsGATA7* modulated some BR‐mediated growth regulations, and *OsGATA7* also had BR‐independent regulation in plant growth, especially in reproductive growth. AM and CRI lines are suitable for dense planting because of the ideal architecture, especially CRI lines (more compact than AM lines). The enhanced plant number in the same area, and no severe reduced grain weight and number in individual plant, increases the grain yield in the unit area obviously. And so far we did not observe that the AM and CRI1 lines are more sensitive to pathogen/herbivore or abiotic stress, which mean they would be putative candidate lines for breeding. Taken together, our work identified *OsGATA7* functioning in BR‐mediated architecture regulation, panicle development and grain shape/number/weight/yield. *OsGATA7* modulates BR‐mediated rice growth regulation and avoids side‐effects of BR signalling, which would be a putative candidate gene for potentially using in agriculture production as *OsGATA7* knock‐down/genome‐edited lines have ideal architecture, better grain shape, and enhanced grain yield.

## References

[pbi12887-bib-0001] Hudson, D. , Guevara, D.R. , Hand, A.J. , Xu, Z. , Hao, L. , Chen, X. , Zhu, T. *et al* (2013) Rice cytokinin GATA transcription Factor1 regulates chloroplast development and plant architecture. Plant Physiol. 162, 132–144. 23548780 10.1104/pp.113.217265 PMC3641198

[pbi12887-bib-0002] Reyes, J.C. , Muro‐Pastor, M. and Florencio, F.J. (2004) The GATA family of transcription factors in Arabidopsis and rice. Plant Physiol. 134, 1718–1732. 15084732 10.1104/pp.103.037788 PMC419845

[pbi12887-bib-0003] Sumiyo, T. , Motoyuki, A. , Shozo, F. , Suguru, T. , Shigeo, Y. , Masahiro, Y. , Atsushi, Y. *et al* (2005) A novel cytochrome P450 is implicated in brassinosteroid biosynthesis via the characterization of a rice dwarf mutant, *dwarf11*, with reduced seed length. Plant Cell, 17, 776–790. 15705958 10.1105/tpc.104.024950 PMC1069698

[pbi12887-bib-0004] Wang, L. , Yin, H. , Qian, Q. , Yang, J. , Huang, C. , Hu, X. and Luo, D. (2009) *NECK LEAF 1*, a GATA type transcription factor, modulates organogenesis by regulating the expression of multiple regulatory genes during reproductive development in rice. Cell Res. 19, 598–611. 19337211 10.1038/cr.2009.36

[pbi12887-bib-0005] Yamamuro, C. , Ihara, Y. , Wu, X. , Noguchi, T. , Fujioka, S. , Takatsuto, S. , Ashikari, M. *et al* (2000) Loss of function of a rice brassinosteroid insensitive1 homolog prevents internode elongation and bending of the lamina joint. Plant Cell, 12, 1591–1606. 11006334 10.1105/tpc.12.9.1591 PMC149072

